# Failure of Hepatic Insulin Clearance via CEACAM1-Mediated Endocytosis: The Missing Physiological Link Between Proinsulin Misfolding, Hyperinsulinemia, and Metabolic Complications in Type 2 Diabetes, Obesity, and NAFLD

**DOI:** 10.14341/probl13658

**Published:** 2026-05-20

**Authors:** Maher Monir Akl, Amr Ahmed

**Affiliations:** National Research Lobachevsky State University of Nizhny Novgorod; Faculty of Medicine, National Research Lobachevsky State University of Nizhny Novgorod; The public health department, Riyadh First Health Cluster, Ministry of Health; The public health department, Riyadh First Health Cluster, Ministry of Health

**Keywords:** Hepatic Insulin Clearance, CEACAM1 Endocytosis, Proinsulin Misfolding, Hyperinsulinemia, Obesity, Non-Alcoholic Fatty Liver Disease, Hepatic Insulin Clearance, CEACAM1 Endocytosis, Proinsulin Misfolding, Hyperinsulinemia, Obesity, Non-Alcoholic Fatty Liver Disease

## Abstract

Type 2 diabetes mellitus (T2DM), projected to affect 700 million individuals by 2045, may be driven by a hypothesized physiological axis where impaired hepatic insulin clearance, mediated by CEACAM1 endocytosis, links proinsulin misfolding to chronic hyperinsulinemia, exacerbating T2DM, obesity, and non-alcoholic fatty liver disease (NAFLD). Approximately 50–80% of proinsulin, synthesized at ~6000 molecules per second, undergoes 5–10% misfolding due to disrupted disulfide bonds (B7-A7, B19-A20, A6-A11) under endoplasmic reticulum (ER) stress, compounded by glutathione (GSH) depletion, which primarily impairs protein disulfide isomerase (PDI) function critical for insulin synthesis. Hepatic clearance involves CEACAM1 binding, insulin receptor isoform B (IR-B) tyrosine 960 autophosphorylation, AP-2/clathrin/dynamin vesicle formation, Rab5-mediated acidification (pH 5.5), Rab7 trafficking, and lysosomal cathepsin B/D hydrolysis, supported by IR-B–IRS-1 tyrosine 608–PI3K–PDK1–Akt (Ser473)–GSK3β signaling. ER stress activates the unfolded protein response (UPR: IRE1α-XBP1, PERK-eIF2α, ATF6), increasing clearance demand.

When clearance fails, misfolded proinsulin accumulates, driving hyperinsulinemia, which promotes insulin resistance, activates mTORC1–SREBP-1c-mediated lipogenesis and VLDL export, and contributes to NAFLD and obesity, as observed in CEACAM1 knockout models. The Hepatic Insulin Clearance Index (HICI), derived from a 50-g glucose challenge yielding a C-peptide/insulin ratio<1, may diagnose impaired clearance with greater sensitivity than HOMA-IR. Emerging tools, such as cryo-EM and portal vein proteomics, could quantify misfolded aggregates and elucidate NAFLD connections. Potential therapeutic strategies, including CEACAM1 enhancers and IR-B agonists, may target this clearance axis. This hypothesis underscores hepatic insulin clearance as a potential mediator of insulin resistance and its role in diseases linked to chronic hyperinsulinemia.

## 1. INTRODUCTION

Type 2 diabetes mellitus (T2DM) represents a global health crisis, projected to affect 700 million individuals by 2045, with its pathogenesis intricately linked to hyperinsulinemia, obesity, and non-alcoholic fatty liver disease (NAFLD) [1]. At the core of this metabolic dysregulation lies the hepatic insulin clearance mechanism, a critical process mediated by the liver to regulate systemic insulin levels [2]. Proinsulin, synthesized within the endoplasmic reticulum (ER) of pancreatic beta-cells at a rate of approximately 6000 molecules per second under metabolic demand, undergoes a complex folding process involving the formation of intra- and inter-chain disulfide bonds (B7-A7, B19-A20, A6-A11) facilitated by binding immunoglobulin protein (BiP) and protein disulfide isomerase (PDI) [3].

However, under conditions of endoplasmic reticulum stress, approximately 5-10% of proinsulin molecules exhibit misfolding, forming intermolecular aggregates due to incorrect disulfide pairing, which triggers the unfolded protein response (UPR) [4]. This response encompasses IRE1α oligomerization leading to XBP1 mRNA splicing, PERK autophosphorylation resulting in eIF2α phosphorylation, and ATF6 translocation to the Golgi for cleavage and nuclear translocation, collectively aiming to restore ER homeostasis but potentially leading to beta-cell apoptosis if unresolved [5][6]. Following secretion, proinsulin and mature insulin traverse the portal vein to the liver, where concentrations are significantly elevated compared to peripheral circulation [7]. Here, hepatic insulin clearance initiates through the binding of proinsulin or insulin to the extracellular immunoglobulin-like domains of CEACAM1 on hepatocyte surfaces, a process enhanced by insulin receptor subtype B (IR-B) autophosphorylation at tyrosine 960 [8].

This phosphorylation event recruits adaptor protein 2 (AP-2), initiating clathrin triskelion assembly into coated pits, followed by dynamin GTP hydrolysis to facilitate vesicle scission [9].

The internalized complex progresses through Rab5-mediated early endosome acidification (pH 5.5 via V-ATPase), where proinsulin dissociates, and subsequently Rab7-mediated late endosome trafficking leads to lysosomal fusion via LAMP1 [10]. Within the lysosome, cathepsin B cleaves at hydrophobic residues (Phe, Tyr, Leu), while cathepsin D targets acidic residues (Asp, Glu), achieving up to 80% degradation, with insulin degrading enzyme (IDE) contributing to residual peptide hydrolysis [11][12]. Concurrently, IR-B signaling amplifies this process through tyrosine 960 docking of IRS-1, leading to tyrosine 608 phosphorylation, PI3K p85-p110 activation, PIP3 production, PDK1 recruitment, Akt serine 473 phosphorylation, and GSK3β serine 9 phosphorylation, which inhibits glycogenolysis and gluconeogenesis while regulating lipogenesis to mitigate NAFLD [13].

The failure of this clearance mechanism precipitates hyperinsulinemia, driving adipose tissue expansion (obesity) and hepatic triglyceride accumulation (NAFLD) via sterol regulatory element-binding protein 1c (SREBP-1c) activation and increased very-low-density lipoprotein (VLDL) export [14]. This study posits that proinsulin misfolding under ER stress, exacerbated by UPR, is a natural consequence of beta-cell overload, and hepatic clearance serves as a protective filter. The objective herein is to elucidate the molecular pathways of hepatic insulin clearance, assess its role in preventing hyperinsulinemia, obesity, and NAFLD, and propose the Hepatic Insulin Clearance Index (HICI) as a diagnostic tool, setting the stage for targeted therapeutic interventions.

## 2. MOLECULAR MECHANISMS OF HEPATIC INSULIN CLEARANCE

The molecular orchestration of hepatic insulin clearance commences with proinsulin biosynthesis within the ER of pancreatic beta-cells. Preproinsulin is translated, its signal peptide cleaved, and the resulting 86-amino-acid proinsulin stabilized by BiP, which facilitates the formation of three disulfide bonds: intra-chain B7-A7, inter-chain B19-A20, and intra-chain A6-A11 [15]. Under metabolic stress, with a production rate of 6000 molecules per second, ER overload leads to misfolding in 5-10% of proinsulin molecules, characterized by incorrect disulfide pairing and intermolecular aggregate formation [16]. This misfolded proinsulin is processed through the Golgi apparatus, packaged into secretory granules, and converted to mature insulin before secretion into the portal vein, where concentrations are markedly higher than in systemic circulation [17].

Upon reaching the liver, proinsulin and insulin engage the CEACAM1 receptor on hepatocyte membranes via its extracellular immunoglobulin-like domains, binding preferentially to the B-chain of proinsulin. This interaction is potentiated by IR-B, which undergoes autophosphorylation at tyrosine 960, triggering CEACAM1 tyrosine phosphorylation. This recruits AP-2 adaptor proteins, initiating clathrin triskelion assembly into coated pits on the plasma membrane [18][19].

Dynamin GTP hydrolysis then drives vesicle scission, internalizing the complex into the endocytic pathway [20]. The vesicle matures into an early endosome under Rab5 GTPase control, where V-ATPase-mediated acidification to pH 5.5 facilitates proinsulin dissociation from CEACAM1, aided by EEA1 tethering [21]. Progression to late endosomes, governed by Rab7 GTPase, culminates in SNARE-mediated fusion with LAMP1-positive lysosomes [22]. Within this compartment, cathepsin B, an endopeptidase, cleaves at hydrophobic sites (phenylalanine, tyrosine, leucine), while cathepsin D, an aspartic protease, targets acidic residues (aspartate, glutamate), achieving approximately 80% degradation of proinsulin/insulin [23]. Insulin degrading enzyme (IDE) in the cytosol further hydrolyzes residual peptides, with an estimated 20% chain dissociation into A and B fragments [24].

Parallel to this degradative pathway, IR-B signaling enhances clearance efficiency. Autophosphorylation at tyrosine 960 creates a docking site for IRS-1 and Shc, leading to IRS-1 tyrosine 608 phosphorylation [25]. This recruits the PI3K p85 regulatory subunit, activating the p110 catalytic subunit to produce PIP3, a second messenger that recruits PDK1 via its pleckstrin homology domain [26]. PDK1 phosphorylates Akt at threonine 308 and serine 473, with the latter inhibiting GSK3β via serine 9 phosphorylation [27]. This cascade suppresses glycogenolysis and gluconeogenesis while modulating lipogenesis, preventing hepatic steatosis and adipose expansion.

The link to obesity and NAFLD emerges from clearance failure, where persistent hyperinsulinemia activates mTORC1, upregulating SREBP-1c to enhance lipogenesis and VLDL export, driving hepatic triglyceride accumulation and adipocyte hypertrophy [28]. ER stress-induced misfolding, mitigated by UPR (IRE1α endonuclease activity splicing XBP1u to XBP1s, PERK autophosphorylation inhibiting eIF2α translation, ATF6 translocation inducing HSP70), is a natural response to beta-cell overload [29][30]. However, if UPR fails, misfolded proinsulin accumulates, necessitating hepatic clearance as a systemic safeguard. A secondary consideration involves glutathione (GSH) depletion impairing PDI, potentially disrupting disulfide bond formation, though this effect is context-dependent and not the primary driver of clearance efficacy [31][32].

## 3. HYPOTHESIS OF HEPATIC INSULIN CLEARANCE: MECHANISTIC FRAMEWORK

The hypothesis posits that the hepatic insulin clearance mechanism, mediated by CEACAM1-dependent endocytosis, serves as a pivotal regulator in mitigating the systemic consequences of proinsulin misfolding, thereby preventing the progression to hyperinsulinemia, obesity, and non-alcoholic fatty liver disease (NAFLD) in type 2 diabetes (T2DM). This framework delineates a dynamic interplay wherein proinsulin, post-secretion from beta-cells, enters the portal vein and engages hepatocytes through a highly orchestrated molecular cascade.

The process initiates with CEACAM1 binding to proinsulin’s B-chain, synergistically enhanced by IR-B autophosphorylation at tyrosine 960, which activates a downstream signaling network [33]. This network recruits AP-2 adaptor complexes, facilitating clathrin triskelion polymerization into coated pits, with dynamin GTP hydrolysis mediating vesicle scission to form early endosomes. Rab5 GTPase drives endosomal acidification to pH 5.5, promoting proinsulin dissociation, followed by Rab7 GTPase-directed trafficking to late endosomes, culminating in SNARE-mediated fusion with LAMP1-positive lysosomes [34]. Here, cathepsin B cleaves at aliphatic residues, while cathepsin D targets polar acidic sites, achieving efficient degradation, supplemented by insulin degrading enzyme (IDE) activity on residual peptides [35].

Concurrently, IR-B signaling amplifies clearance through tyrosine 960 docking IRS-1, triggering tyrosine 608 phosphorylation, which recruits PI3K’s p85 subunit to activate the p110 catalytic domain, generating PIP3 [36]. This recruits PDK1, phosphorylating Akt at threonine 308 and serine 473, subsequently inhibiting GSK3β via serine 9 phosphorylation [37]. This inhibition suppresses gluconeogenic enzymes PEPCK and G6Pase, stabilizing glucose homeostasis and modulating lipid metabolism to avert NAFLD [38]. The hypothesis further suggests that failure of this clearance pathway, due to impaired CEACAM1 expression or endosomal dysfunction, results in proinsulin accumulation, driving hyperinsulinemia [39][40].

This excess insulin activates mTORC1, upregulating SREBP-1c to enhance de novo lipogenesis and VLDL secretion, fostering hepatic steatosis and adipocyte hypertrophy, hallmarks of NAFLD and obesity [41]. The mechanistic framework also incorporates the role of endoplasmic reticulum (ER) stress as a precursor to clearance demand. Under beta-cell stress, misfolded proinsulin aggregates trigger the unfolded protein response (UPR), characterized by IRE1α-mediated activation of ASK1-JNK, phosphorylating IRS-1 at serine 307 to inhibit insulin signaling, PERK-induced eIF2α phosphorylation to attenuate protein synthesis, and ATF6 translocation to induce chaperone expression [42]. While UPR attempts to resolve ER stress, its failure escalates proinsulin burden, necessitating robust hepatic clearance. A secondary consideration involves glutathione (GSH) depletion potentially impairing PDI-mediated disulfide bond isomerization, though this is posited as a modulating factor rather than a primary determinant [43]. The hypothesis predicts that enhancing CEACAM1-mediated clearance could reverse hyperinsulinemia-driven metabolic dysfunction, with therapeutic implications for obesity and NAFLD, warranting further investigation into endosomal flux and lysosomal capacity.

## 4. TARGETING HEPATIC INSULIN CLEARANCE: THERAPEUTIC STRATEGIES

A primary approach involves augmenting CEACAM1 expression to optimize proinsulin uptake, initiating the endocytic pathway with AP-2 recruitment, clathrin polymerization, and dynamin-driven vesicle scission. This enhances Rab5-mediated early endosome formation and Rab7-directed trafficking to lysosomes, where cathepsin B and D activities, alongside insulin degrading enzyme (IDE), are potentiated to achieve near-complete proinsulin hydrolysis. Pharmacological agents that stabilize Rab7 GTPase activity or upregulate LAMP1 expression could further bolster lysosomal fusion efficiency, critical for degrading misfolded proinsulin aggregates [44].

Parallel strategies focus on reinforcing IR-B signaling to amplify clearance efficacy. Agonists targeting tyrosine 960 autophosphorylation could enhance IRS-1 tyrosine 608 phosphorylation, activating the PI3K p85-p110 complex to produce PIP3, recruiting PDK1, and phosphorylating Akt at serine 473 [45]. This cascade inhibits GSK3β, reducing gluconeogenesis and lipogenesis mediated by SREBP-1c, thereby mitigating hepatic steatosis and adipose tissue expansion linked to obesity and NAFLD [46]. Additionally, modulating ER stress responses offers a complementary avenue; inhibitors of IRE1α-ASK1-JNK signaling or enhancers of ATF6-induced chaperone expression could alleviate UPR burden, decreasing proinsulin misfolding and the subsequent demand on hepatic clearance [47].

## 5. MECHANISTIC SUPPORTING EVIDENCE FOR HEPATIC INSULIN CLEARANCE AS A PHYSIOLOGICAL MEDIATOR

## 5.1. Physiological necessity and first-pass control

Hepatic insulin clearance (HIC) via CEACAM1 emerges as a built-in “quality-and-quantity” gate that determines how much and what form of secreted insulin reaches the systemic circulation. Genetic disruption of CEACAM1’s phosphorylation in the liver impairs receptor-mediated endocytosis and lysosomal degradation of insulin, producing primary hyperinsulinemia that precedes and drives insulin resistance, visceral adiposity, and hepatic lipid accumulation. This establishes HIC failure as an upstream lesion rather than a bystander to insulin resistance [53].

## 5.2. Bidirectional causality with diet and restoration by rescue

Nutritional stress such as high-fat feeding lowers hepatic CEACAM1 by more than 50% within weeks, reducing HIC and triggering hyperinsulinemia, insulin resistance, and hepatic triacylglycerol accumulation (table 1) [54]. Conversely, inducible, liver-specific CEACAM1 re-expression restores HIC, prevents hyperinsulinemia, and limits insulin resistance and steatosis demonstrating causality and reversibility at the organ level [55]. Mechanistically, CEACAM1 phosphorylation downstream of IR-B promotes adaptor (AP-2) recruitment, clathrin assembly, dynamin scission, Rab5/Rab7 endosomal trafficking, and lysosomal cathepsin-dependent proteolysis, thereby accelerating removal of inactive or aberrant insulin species during the first pass [56].

**Table:** Table 1. Summary of proposed therapeutic strategies to enhance hepatic insulin clearance. The interventions target endocytic trafficking, insulin receptor signaling, ER stress regulation, and diagnostic monitoring, aiming to reduce hyperinsulinemia, prevent obesity and NAFLD, and enable personalized therapeutic approaches.

Therapeutic Strategy	Molecular Target	Mechanism of Action	Expected Outcome
CEACAM1 Enhancers	CEACAM1, AP-2, Clathrin, Dynamin	Increases endocytic uptake and vesicle scission	Enhanced proinsulin clearance, reduced hyperinsulinemia [48]
Rab7/LAMP1 Stabilizers	Rab7 GTPase, LAMP1	Promotes late endosome–lysosome fusion	Improved lysosomal degradation, NAFLD mitigation [49]
IR-B Agonists	IR-B (Tyr960), IRS-1, PI3K, Akt, GSK3β	Activates signaling cascade to inhibit gluconeogenesis	Reduced lipogenesis, obesity/NAFLD prevention [50]
IRE1α/ATF6 Modulators	IRE1α-ASK1-JNK, ATF6	Regulates UPR to reduce misfolding	Decreased ER stress, lower clearance demand [51]
HICI Monitoring	Proinsulin, Insulin, C-peptide ratios	Assesses clearance efficiency via glucose challenge	Personalized therapy, early NAFLD detection [52]

## 5.3. Lipid signaling cross-talk: the insulin–CEACAM1–FASN axis

Beyond endocytosis, internalized CEACAM1 couples to fatty acid synthase (FASN), acutely down-modulating lipogenesis under physiological insulin pulses. When CEACAM1 is reduced, this brake is lifted: chronic hyperinsulinemia sustains mTORC1–SREBP-1c activation, boosts de novo lipogenesis and VLDL export, and accelerates steatosis. Thus, impaired HIC simultaneously elevates circulating insulin, driving receptor desensitization, and disinhibits hepatocellular lipogenesis, forming a self-reinforcing loop that links clearance failure to NAFLD biology [57][58].

## 5.4. Cell-type specificity and fibrogenic progression

Conditional hepatic deletion pinpoints hepatocytes as the critical compartment: loss of CEACAM1 in hepatocytes reduces HIC, causing hyperinsulinemia-driven hepatic insulin resistance, steatohepatitis, and progression to fibrosis even on standard chow. In contrast, endothelial deletion promotes inflammation-driven fibrosis without metabolic derailment, separating fibrogenic pathways from clearance-driven metabolic injury. These in vivo dissections cement impaired hepatocyte HIC as a proximate driver of chronic hyperinsulinemia and its steato-fibrotic sequelae [59].

## 5.5. Human translational concordance

Clinical data mirror the preclinical findings: individuals with metabolic dysfunction–associated steatohepatitis exhibit a progressive decline of hepatic CEACAM1 across hepatocytes and sinusoidal endothelium as fibrosis advances, consistent with a graded loss of first-pass control. Reduced hepatic CEACAM1 expression is observed in individuals with insulin resistance, obesity, and fatty liver disease, underscoring its relevance to human pathology [60].

## 5.6. Etiologic framing of type 2 diabetes

Synthesis across genetics, nutrition, hepatocyte biology, and human data supports an etiologic model in which reduced HIC is sufficient to produce the hyperinsulinemic milieu that seeds insulin resistance and hepato-metabolic remodeling. Lower insulin clearance can act as a primary cause of type 2 diabetes in susceptible individuals aligning with the mechanistic cascade above and positioning hepatic clearance as the physiological mediator that links upstream molecular derangements to systemic metabolic disease [61].

## 5.7. Pathway synthesis for the hypothesis

Under β-cell stress, a fraction of proinsulin is vulnerable to misfolding and redox-dependent modifications; the liver’s CEACAM1-directed endocytic–lysosomal system is evolutionarily poised to “scrub” these species during the portal first pass. When CEACAM1 is diminished, this scrubber fails, allowing spillover of aberrant or less-active insulin into the periphery while simultaneously amplifying hepatocellular lipogenesis through loss of CEACAM1–FASN regulation.

Together, these processes drive chronic hyperinsulinemia, receptor desensitization due to impaired IR-B/IRS-1/PI3K/Akt/GSK3β signaling, and progression of NAFLD.This integrative mechanism positions hepatic insulin clearance as the physiological mediator that converts upstream insulin misfolding and redox instability into downstream chronic hyperinsulinemia, insulin resistance, lipogenesis, steatohepatitis, and fibrosis [62][63].

## 6. PROPOSED DIAGNOSTIC TOOL: HEPATIC INSULIN CLEARANCE INDEX (HICI)

The Hepatic Insulin Clearance Index (HICI) is introduced as a refined diagnostic and monitoring tool to evaluate hepatic insulin clearance efficiency in T2DM, providing insights into hyperinsulinemia, obesity, and NAFLD progression. This index is determined through a 50-gram oral glucose tolerance test, with plasma proinsulin, insulin, and C-peptide levels assessed at intervals (0, 30, 60, 90, 120 minutes). The C-peptide to insulin ratio is the key indicator: a ratio exceeding 2 denotes normal clearance, whereas a ratio below 1 signifies impairment, associated with elevated proinsulin and hyperinsulinemia, serving as a predictor for NAFLD and obesity.

At the molecular level, HICI mirrors the efficacy of CEACAM1-mediated endocytosis, where tyrosine-phosphorylated CEACAM1 recruits AP-2 and clathrin, driving dynamin-mediated vesicle formation, followed by Rab5 acidification and Rab7 trafficking to lysosomes for cathepsin-driven degradation. Simultaneously, IR-B signaling, initiated by tyrosine 960 autophosphorylation, enhances this process via IRS-1, PI3K, and Akt activation, regulating hepatic glucose and lipid metabolism. A reduced HICI ratio suggests dysfunction, potentially from diminished CEACAM1 expression or lysosomal inefficiency, leading to proinsulin accumulation that activates SREBP-1c-driven lipogenesis and VLDL export, worsening NAFLD [64][65][66].

HICI offers superior specificity over HOMA-IR by targeting liver-specific clearance, with initial data suggesting its efficacy in identifying early metabolic decline in obese individuals. However, its clinical adoption necessitates validation through larger studies to define threshold values and correlate with NAFLD severity. Integration into clinical practice could enable tailored interventions, though further optimization is required to address interindividual variations in endosomal function.

The HICI ratio is computed by dividing the area under the curve (AUC) for C-peptide concentrations by the AUC for insulin concentrations over the 120-minute period, derived from ELISA measurements.

This ratio reflects the liver’s ability to clear insulin relative to its secretion, with a threshold of >2 indicating normal hepatic function and <1 suggesting impairment. Validation involves duplicate assays to ensure reproducibility, followed by comparison with gold-standard methods like hyperinsulinemic-euglycemic clamp studies in a subset of patients. Statistical analysis, including receiver operating characteristic (ROC) curves, is employed to determine sensitivity and specificity, establishing diagnostic cutoffs. Results are corroborated through longitudinal tracking of NAFLD markers (e.g., liver fat content via MRI) and obesity indices (e.g., BMI, waist circumference) to confirm predictive accuracy. Diagnosis is confirmed when a persistent HICI <1 aligns with clinical signs of hyperinsulinemia, elevated liver enzymes, or imaging evidence of hepatic steatosis, guiding personalized therapeutic strategies.

## 7. FUTURE DIRECTIONS AND THERAPEUTIC PROSPECTS

Future advancements in hepatic insulin clearance for T2DM aim to deepen molecular understanding and enhance therapeutic outcomes for hyperinsulinemia, obesity, and NAFLD. Cryo-electron microscopy (cryo-EM) is proposed to map the structural interactions of CEACAM1, dynamin, and clathrin during vesicle formation, offering detailed insights into endocytic mechanics.

Non-reducing mass spectrometry of portal vein samples could quantify proinsulin aggregates, providing direct evidence of their role in NAFLD development.

Therapeutically, compounds enhancing CEACAM1 expression are envisioned to boost AP-2 recruitment and clathrin polymerization, improving endocytic uptake.

IR-B agonists targeting tyrosine 960 autophosphorylation could enhance IRS-1 tyrosine 608 phosphorylation, activating PI3K-p110 to produce PIP3, recruiting PDK1, and phosphorylating Akt to inhibit GSK3β, thereby reducing gluconeogenesis and lipogenesis in NAFLD. Modulators of Rab7 GTPase or LAMP1 expression are suggested to enhance late endosome-lysosome fusion, optimizing cathepsin-mediated degradation. In obesity models, CEACAM1 knockout combined with dietary challenges could evaluate clearance restoration’s effect on adipocyte growth.

The Hepatic Insulin Clearance Index (HICI) is poised to guide these interventions, enabling real-time clearance assessment and personalized adjustments, especially in obese patients where adipose tissue amplifies insulin resistance. Clinical trials are recommended to test endosomal flux enhancers and IR-B agonists in NAFLD cohorts, with outcomes including liver fat reduction and insulin sensitivity improvement (table 2 & table 3).

**Table:** Table 2. Summarizes the methodological pipeline for calculating the Hepatic Insulin Clearance Index (HICI), from glucose challenge through biomarker quantification and ratio analysis. The integration of proinsulin, insulin, and C-peptide dynamics provides a direct measure of hepatic clearance efficiency. Diagnostic thresholds C-peptide/insulin ratio >2 indicating normal clearance and <1 indicating impairment are linked to hyperinsulinemia, obesity, and NAFLD progression. This structured framework highlights HICI’s potential as a liver-specific diagnostic tool, offering both clinical utility and translational relevance in metabolic disease.

Step	Methodology	Molecular Focus	Expected Outcome	Time Points (min)
1. Glucose Challenge	Oral administration of 50 g glucose	Stimulates insulin/proinsulin secretion	Initiates beta-cell response	0
2. Blood Sampling	Venipuncture for plasma collection	Captures proinsulin, insulin, C-peptide	Provides baseline and dynamic data	0, 30, 60, 90, 120
3. Assay Execution	ELISA for proinsulin, insulin, C-peptide	Quantifies peptide levels	Measures clearance efficiency	Post-collection
4. Ratio Calculation	C-peptide/insulin ratio computation	Assesses hepatic clearance capacity	Ratio >2 (normal), <1 (impaired)	Post-assay
5. Correlation Analysis	Statistical analysis vs. NAFLD/obesity	Links ratio to metabolic markers	Predicts disease progression	Post-calculation

**Table:** Table 3. Outlines prospective experimental and therapeutic directions designed to advance the understanding and clinical translation of hepatic insulin clearance. Approaches such as cryo-EM imaging and portal proteomics aim to provide structural and biochemical validation of clearance pathways, while therapeutic candidates CEACAM1 enhancers, IR-B agonists, and Rab7/LAMP1 modulators focus on optimizing endocytosis, signaling, and lysosomal function. The inclusion of HICI-guided trials emphasizes personalized medicine, linking mechanistic insights to tailored therapeutic interventions in NAFLD and T2DM.

Research/Therapeutic Approach	Target/Technique	Mechanism/Methodology	Anticipated Outcome
Cryo-EM Imaging	CEACAM1-Dynamin-Clathrin	High-resolution structural analysis	Elucidates endocytic complex dynamics
Portal Proteomics	Proinsulin Aggregates	Non-reducing mass spectrometry	Quantifies NAFLD-linked aggregates
CEACAM1 Enhancers	CEACAM1, AP-2, Clathrin	Increases endocytic uptake	Enhanced clearance, reduced hyperinsulinemia
IR-B Agonists	IR-B (Tyr960), Akt, GSK3β	Activates signaling to inhibit lipogenesis	NAFLD and obesity mitigation
Rab7/LAMP1 Modulators	Rab7, LAMP1	Enhances lysosomal fusion	Improved degradation efficiency
HICI-Guided Trials	Clearance Efficiency	Glucose challenge with ratio monitoring	Personalized therapy, NAFLD prevention

## 8. DISCUSSION

The very existence of hepatic insulin clearance as a robust physiological process strongly supports the premise that insulin and proinsulin molecules, particularly under metabolic pressure, are susceptible to misfolding and structural distortion [67]. If every insulin molecule secreted were perfectly folded and functionally intact, there would be little evolutionary need for a clearance system capable of degrading up to 80% of portal insulin on first pass [68]. The presence of CEACAM1-mediated clearance therefore indicates that the organism anticipates structural imperfections, and has evolved a mechanism to selectively remove misfolded or chemically unstable molecules before they reach the systemic circulation [69]. This physiological safeguard is itself powerful indirect evidence that proinsulin misfolding is not a rare aberration, but a recurrent outcome of β-cell stress that requires hepatic protection to preserve systemic homeostasis [70]. At the molecular level, sulfur metabolism lies at the origin of this process. Adequate sulfur supply ensures sufficient glutathione (GSH) synthesis, which in turn maintains protein disulfide isomerase (PDI) activity in the endoplasmic reticulum (ER) [71]. PDI is critical for reshuffling cysteine residues and establishing correct disulfide bonds in proinsulin [72].

Sulfur deficiency reduces GSH pools and impairs PDI function, destabilizing key disulfide linkages (A6–A11, A7–B7, A20–B19) and generating a fraction of misfolded proinsulin [73]. This burden activates the unfolded protein response (UPR) via IRE1α–XBP1 splicing, PERK–eIF2α phosphorylation, and ATF6 signaling, reflecting β-cell stress and an attempt to restore proteostasis [74]. Yet, under chronic pressure, up to 5–10% of the ~6000 molecules synthesized per second are misfolded, exceeding the ER’s corrective capacity [3].

Upon secretion, these misfolded molecules enter the portal circulation, where they encounter an environment dominated by hepatic redox biology. Insulin disulfide bonds remain labile outside the cell, vulnerable to thiol–disulfide exchange. Chain-splitting occurs when GSH or other thiols attack disulfide bridges, cleaving the insulin molecule and reducing its bioactivity [75]. Rates of chain-splitting can approach ~20%, depending on the prevailing redox potential. Since the liver is the major source of circulating GSH, hepatic sulfur metabolism directly governs extracellular insulin stability [75]. When GSH levels are high and the redox potential is low, chain cleavage accelerates; when GSH is depleted, chain-splitting slows and insulin remains more stable [76]. This redox-driven instability extends the sulfur hypothesis beyond the ER, showing that insulin vulnerability continues into the vascular compartment [73]. Hepatic insulin clearance integrates these challenges through CEACAM1-mediated endocytosis, providing the physiological checkpoint that determines whether misfolded or unstable insulin molecules are neutralized or allowed to persist systemically.

Mechanistically, insulin or proinsulin binding to insulin receptor-B (IR-B) induces Tyr960 autophosphorylation, promoting CEACAM1 phosphorylation and recruitment of AP-2 [77]. This initiates clathrin-coated pit formation, dynamin-mediated vesicle scission, and trafficking through Rab5-positive early endosomes with acidification to pH ~5.5. Endosomes then mature into Rab7-positive compartments that fuse with lysosomes, where cathepsin B and D hydrolyze insulin peptides. Approximately 50–80% of portal insulin undergoes degradation by this route in the first hepatic pass, ensuring that only structurally intact insulin achieves systemic circulation.

When sulfur deficiency increases misfolding at the β-cell level and GSH-dependent chain-splitting destabilizes insulin in plasma, clearance demand surges. CEACAM1 expression or function may become saturated or impaired, and the endocytic machinery overwhelmed. This failure allows spillover of misfolded, chain-split, or otherwise defective insulin into the systemic circulation. β-cells, sensing reduced signaling efficiency, compensate with increased secretion, resulting in chronic hyperinsulinemia. Elevated insulin levels, many molecules of which are structurally compromised, engage IR-B and IRS-1 inefficiently, disrupting PI3K–PIP3–PDK1–Akt signaling and further aggravating insulin resistance [75].

In parallel, chronic hyperinsulinemia activates mTORC1–SREBP-1c signaling, driving de novo lipogenesis and very-low-density lipoprotein (VLDL) export, which link the clearance defect directly to hepatic steatosis and adipocyte hypertrophy [78][79]. This mechanism explains the well-established association between type 2 diabetes, obesity, and non-alcoholic fatty liver disease (NAFLD) [80].

This clearance-redox framework positions CEACAM1-mediated endocytosis not as a passive degradative pathway, but as the central physiological mediator between molecular misfolding and systemic metabolic disease. Sulfur deficiency produces misfolded substrates, GSH-dependent redox instability drives extracellular chain-splitting, and hepatic clearance integrates both into the outcome of either protection or pathology. If clearance is effective, the system rescues the organism by removing dysfunctional molecules. If clearance fails, the system shifts toward disease, producing chronic hyperinsulinemia as the immediate driver of insulin resistance, hepatic steatosis, and obesity.

## 9. CONCLUSION

Hepatic insulin clearance via CEACAM1-mediated endocytosis may represent the pivotal physiological mediator linking sulfur-dependent proinsulin misfolding and redox-driven chain-splitting to chronic hyperinsulinemia and its complications. In this integrated model, sulfur deficiency impairs PDI-mediated folding, while hepatic GSH availability determines extracellular insulin stability, both converging on clearance efficiency.

When clearance becomes saturated or impaired, defective insulin accumulates systemically, intensifying insulin resistance, promoting steatosis, and accelerating obesity. The Hepatic Insulin Clearance Index (HICI) offers a practical diagnostic metric for clearance dysfunction, while therapeutic strategies targeting CEACAM1 expression, IR-B signaling fidelity, and hepatic redox balance provide rational interventions. If confirmed, this clearance–redox paradigm could redefine type 2 diabetes as a disorder of clearance-mediated hyperinsulinemia superimposed on sulfur-dependent protein misfolding, with direct implications for preventing and treating its global burden.

## ADDITIONAL INFORMATION

Funding Information: No financial support was received for this study.

Competing Interests: The authors declare no conflicts of interest.
